# A randomized, controlled phase II trial of neoadjuvant ado-trastuzumab emtansine, lapatinib, and nab-paclitaxel versus trastuzumab, pertuzumab, and paclitaxel in HER2-positive breast cancer (TEAL study)

**DOI:** 10.1186/s13058-019-1186-0

**Published:** 2019-09-02

**Authors:** Tejal A. Patel, Joe E. Ensor, Sarah L. Creamer, Toniva Boone, Angel A. Rodriguez, Poly A. Niravath, Jorge G. Darcourt, Jane L. Meisel, Xiaoxian Li, Jing Zhao, John G. Kuhn, Roberto R. Rosato, Wei Qian, Anna Belcheva, Mary R. Schwartz, Virginia G. Kaklamani, Jenny C. Chang

**Affiliations:** 1 Houston Methodist Cancer Center, 6445 S. Main St., Houston, TX 77030 USA; 2 Houston Methodist Research Institute, 6670 Bertner Avenue, Houston, TX 77030 USA; 3 The University of Texas Health Science Center at San Antonio, 7703 Floyd Curl Dr., San Antonio, TX 78229 USA; 4 Winship Cancer Institute, Emory University School of Medicine, 1365 Clifton Rd, Atlanta, GA 30322 USA; 5 Affiliated Hospital of Qingdao University, 16 Jiangsu Rd, Shinan Qu, Qingdao Shi, Shandong Sheng China; 6 Department of Pathology and Genomic Medicine, Houston Methodist Hospital, 6565 Fannin St, Houston, TX 77030 USA; 7 Weill Cornell Medicine, 1300 York Avenue, New York, NY 10065 USA

**Keywords:** Neoadjuvant, HER2, Ado-trastuzumab emtansine, T-DM1, Lapatinib, Nab-paclitaxel, RCB

## Abstract

**Background:**

Neoadjuvant dual human epidermal growth factor receptor (HER2) blockade with trastuzumab and pertuzumab plus paclitaxel leads to an overall pathologic complete response (pCR) rate of 46%. Dual HER2 blockade with ado-trastuzumab emtansine (T-DM1) and lapatinib plus nab-paclitaxel has shown efficacy in patients with metastatic HER2-positive breast cancer. To test neoadjuvant effectiveness of this regimen, an open-label, multicenter, randomized, phase II trial was conducted comparing T-DM1, lapatinib, and nab-paclitaxel with trastuzumab, pertuzumab, and paclitaxel in patients with early-stage HER2-positive breast cancer.

**Methods:**

Stratification by estrogen receptor (ER) status occurred prior to randomization. Patients in the experimental arm received 6 weeks of targeted therapies (T-DM1 and lapatinib) followed by T-DM1 every 3 weeks, lapatinib daily, and nab-paclitaxel weekly for 12 weeks. In the standard arm, patients received 6 weeks of trastuzumab and pertuzumab followed by trastuzumab weekly, pertuzumab every 3 weeks, and paclitaxel weekly for 12 weeks. The primary objective was to evaluate the proportion of patients with residual cancer burden (RCB) 0 or I. Key secondary objectives included pCR rate, safety, and change in tumor size at 6 weeks. Hypothesis-generating correlative assessments were also performed.

**Results:**

The 30 evaluable patients were well-balanced in patient and tumor characteristics. The proportion of patients with RCB 0 or I was higher in the experimental arm (100% vs. 62.5% in the standard arm, *p* = 0.0035). In the ER-positive subset, all patients in the experimental arm achieved RCB 0-I versus 25% in the standard arm (*p* = 0.0035). Adverse events were similar between the two arms.

**Conclusion:**

In early-stage HER2-positive breast cancer, the neoadjuvant treatment with T-DM1, lapatinib, and nab-paclitaxel was more effective than the standard treatment, particularly in the ER-positive cohort.

**Trial registration:**

Clinicaltrials.gov NCT02073487, February 27, 2014.

## Background

Human epidermal growth factor receptor (HER2) over-expression is present in about 15–20% of breast cancers [1]. Until the development of HER2-targeted therapies, this breast cancer subtype was associated with a worse prognosis [1]. Despite the success of targeted agents, resistance inevitably develops when these medications are used as monotherapy [2]. Clinically, a more complete blockade of the HER receptor layer has been shown to be therapeutically meaningful in prolonging patient survival [3, 4]. In the NeoALTTO study, dual HER2 blockade with lapatinib and trastuzumab plus paclitaxel resulted in a higher pCR rate when compared with trastuzumab/paclitaxel (51.3% vs. 29.5%) [3]. In the NeoSphere study, dual blockade with pertuzumab and trastuzumab plus docetaxel compared to trastuzumab/docetaxel had a significantly improved pCR rate (46% vs. 29%) [4]. Similar efficacy has been noted regardless of taxane utilized with paclitaxel having an improved side effect profile relative to docetaxel [5]. Thus, the combination of trastuzumab, pertuzumab, and paclitaxel was utilized as the comparator neoadjuvant standard treatment.

In neoadjuvant trials, differences in the rates of pCR have been noted depending on ER status with higher pCR rates in the ER-negative subsets [4, 6]. The pCR response rates were also different in molecularly determined subtypes. The highest pCR rates were in the HER2-enhanced subset while the lowest responses were observed in the HER2-luminal subset [6]. This supports the concept of cross-talk between the HER2 and ER pathways, increasing resistance to HER2-targeted therapies in the HER2-luminal subset. We and others have described activated phosphoinositide 3-kinase (PI3K) pathway (*PIK3CA* mutations or loss of phosphatase and tensin homolog (PTEN)) predicting resistance to trastuzumab [7] but sensitivity to lapatinib [8]. PTEN loss has been described in approximately 50% of breast tumors [9].

Ado-trastuzumab emtansine (T-DM1) is an antibody-drug conjugate in which trastuzumab is bound to DM-1, a taxane-like derivative of maytansine-1 [1, 2]. T-DM1 allows for intracellular drug delivery specifically to HER2-amplified cells. This targeted drug delivery allows for the selective killing of cancer cells, maintaining anti-neoplastic efficacy with an improved side effect profile when compared to routine chemotherapy [10].

Previously, we completed a dose-finding trial of T-DM1, lapatinib, and nab-paclitaxel which yielded a high objective response rate as well as complete responses in heavily pretreated metastatic HER2-positive breast cancer patients [11]. With the high objective response rates in a heavily pretreated metastatic population, we surmised this regimen would be efficacious in early-stage breast cancer as well, allowing benefit in a larger patient population. Previous studies have shown that similar pCR rates are obtained in early-stage, high-risk HER2-positive disease whether paclitaxel or nab-paclitaxel is utilized [12]. Thus, this efficacy was hypothesized to be due to the synergy of HER2-blockade rather than the chemotherapy effect. Building on these earlier studies, a multi-institutional, randomized, phase 2 neoadjuvant clinical trial was carried out by CARE (Consortium for the Advancement of Research Excellence) to test the hypothesis that neoadjuvant dual HER2-targeted therapy with T-DM1, lapatinib, and nab-paclitaxel would yield superior pathologic response when compared with standard neoadjuvant treatment with trastuzumab, pertuzumab, and paclitaxel. Baseline ER status, HER2 subtypes, and PI3K pathway activation were also correlated with pathologic response. Additionally, a 6-week window of targeted therapy alone in both arms was conducted to determine whether changes in tumor sizes on MRI could be a surrogate for subsequent pathologic response.

## Methods

A multicenter, open-label, randomized, phase 2 study was conducted in three institutions of the CARE consortium and was monitored by the institutional Data Safety and Monitoring Board (DSMB) which reviewed adverse events as well as efficacy. This clinical trial (NCT02073487) was conducted in accordance with the Declaration of Helsinki and Good Clinical Practice.

Eligible patients were female, ≥ 18 years of age with adequate performance status and primary tumor ≥ 2 cm in diameter. Patients were required to have histologically confirmed invasive HER2-positive breast cancer which was defined by an immunohistochemical score of 3+, HER2/CEP17 ratio ≥ 2, or average HER2 copy number ≥ 6 [13]. Any nodal status was permitted without metastatic disease. Eligible patients also were required to have left ventricular ejection fraction ≥ 50% as well as adequate bone marrow, kidney, and liver function. Exclusion criteria included separate malignancy < 5 years prior to randomization, pre-existing grade 2+ peripheral neuropathy, uncontrolled serious comorbidities, altered gastrointestinal absorption, pregnant/lactating females, or active infection requiring antibiotics.

**Fig. 1:**
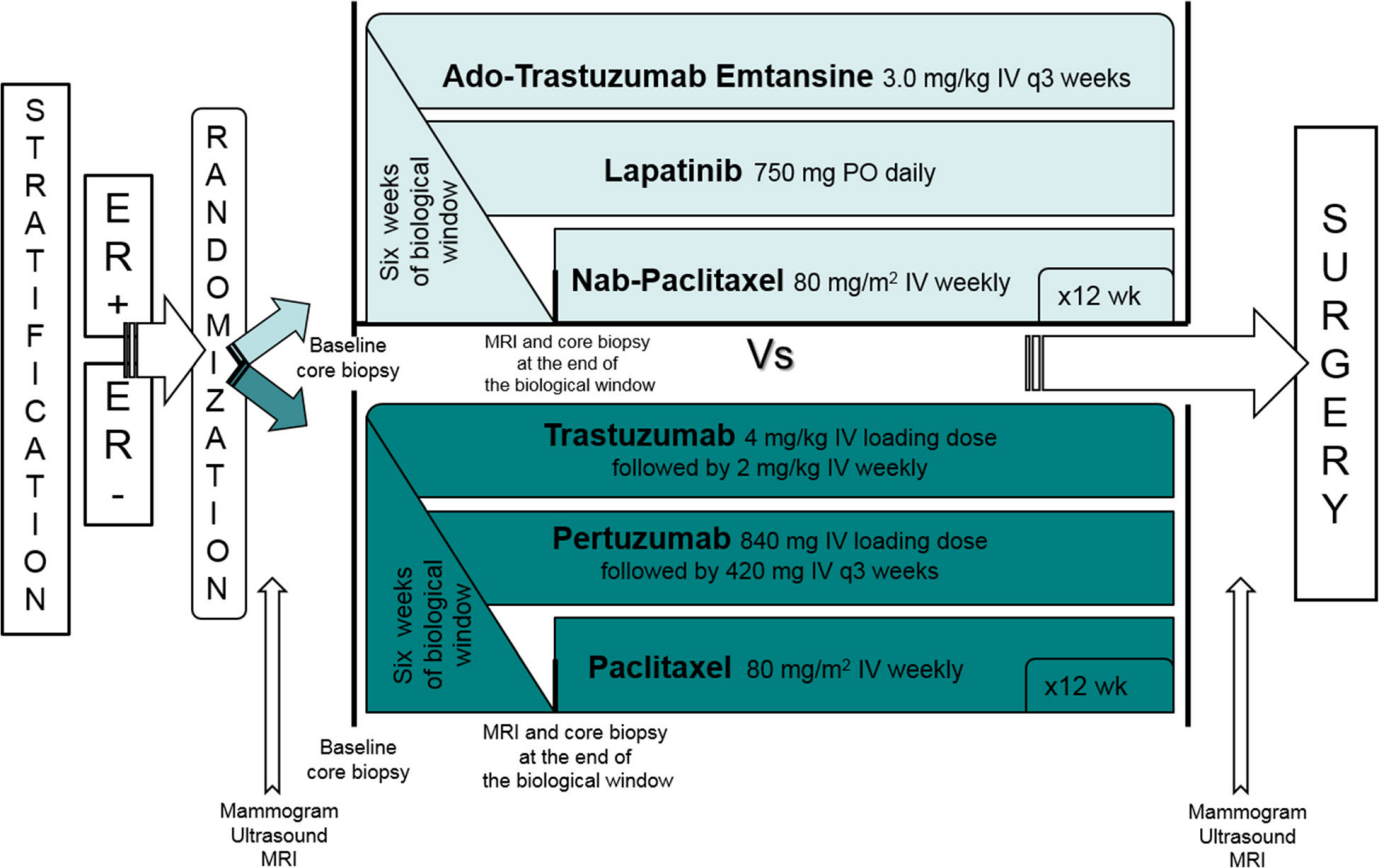
Treatment protocol

After providing informed consent, patients were stratified according to ER status (positive vs. negative) prior to block randomization in groups of 4 to each arm. In the experimental arm, dosing was based on the maximum tolerated dose for this combination as ascertained in the associated phase 1 trial [11]. Patients received a 6-week biologic window of T-DM1 3.0 mg/kg every 3 weeks and lapatinib 750 mg daily followed by continued T-DM1 and lapatinib along with nab-paclitaxel 80 mg/m^2^ weekly for 12 weeks. Loperamide prescription was provided with lapatinib due to high risk of diarrhea. Patients in the standard arm received a 6-week biologic window of trastuzumab and pertuzumab. Loading doses of trastuzumab 4 mg/kg IV and pertuzumab 840 mg IV were followed by subsequent doses of 2 mg/kg IV weekly and 420 mg IV every 3 weeks, respectively. After 6 weeks, paclitaxel 80 mg/m^2^ weekly was added for an additional 12 weeks (Fig. 1). Dose de-escalation for T-DM1 to 2.5 mg/kg, nab-paclitaxel to 70 mg/m^2^, and paclitaxel by 20% was permitted for patients with grade 2+ adverse events. No dose de-escalation was permitted for lapatinib, but it could be held for up to 14 days to allow improvement in grade 2+ adverse events.

Biopsy was performed at baseline and after 6 weeks of targeted therapy. After treatment completion, patients underwent definitive surgery. Baseline biopsies and surgical tissue were placed in formalin for subsequent paraffin embedding or flash frozen on dry ice for later processing. After surgery, decisions regarding adjuvant chemotherapy were left to the treating oncologist. Most patients who did not achieve pCR were treated with subsequent chemotherapy with doxorubicin and cyclophosphamide.

### Efficacy and safety measures

Residual cancer burden (RCB) and pCR were determined from the surgically resected tissue after treatment completion. Magnetic resonance imaging (MRI) was performed at baseline and after 6 weeks of targeted therapy. Patients underwent mammography and breast ultrasound at baseline and before surgery. Hematology and blood chemistry laboratory tests were performed every 3 weeks for 6 weeks and then weekly for 12 weeks. Safety was assessed from the time informed consent was signed through 30 days after the last treatment dose. Adverse events were recorded and graded according to the National Cancer Institute Common Terminology Criteria for Adverse Events (NCI CTCAE) version 4.03.

### Assessing outcomes

The pre-defined primary endpoint was the proportion of patients with residual cancer burden (RCB) 0 or I. RCB was pathologically determined from the (1) tumor bed dimensions, (2) cellularity of the invasive cancer, (3) size of largest nodal metastasis, and (4) number of positive lymph nodes. RCB was categorized as RCB-0 = no residual disease, RCB-I = minimal residual disease, RCB-II = moderate residual disease, and RCB-III = extensive residual disease [14]. RCB-0 was synonymous with pCR, indicating no residual disease present in the breast or axilla. Assessment of RCB and pCR occurred at each affiliated hospital. Key secondary objectives included pCR rate, safety, and change in tumor size by MRI at 6 weeks as previously mentioned.

### Biomarker exploratory studies

Biopsy tissue samples obtained at baseline were used to evaluate biomarkers predictive of response and resistance, including *PIK3CA* mutations, PTEN expression, and HER2 subtypes. Expression of HER2, PTEN, and ER in formalin-fixed, paraffin-embedded (FFPE) tumor tissue samples was evaluated by immunohistochemistry. Transcriptional profiling was performed on mRNA extracted from FFPE samples. HER2 status was evaluated by the HercepTest kit (Dako) and the HER2 IQFISH pharmDx (Dako). ER status was determined by the PharmDx kit (Dako). Genomic analysis of 70 genes to determine molecular subtypes (MammaPrint®) was determined on baseline frozen biopsies (Agendia, Irvine, CA).

To determine the mutational status of *PIK3CA*, DNA was extracted from FFPE slides (Qiagen, Carlsbad, CA) and used to amplify by PCR Exons 9 and 20 (NCBI Reference Sequence: NM_006218.3). Amplified PCR fragments corresponding to Exon 9 (126 bp) and Exon 20 (268 bp) were excised and purified from agarose gel (Thermo Fisher, Waltham, MA) and sequenced (Genewiz, South Plainfield, NJ). Analysis of sequences was performed by using NCBI’s Nucleotide BLAST. Experiments were independently repeated to assure the reproducibility of results.

The assessment of PTEN expression by immunohistochemistry was performed on FFPE specimens that were de-paraffinized. Sections were treated with 3% hydrogen peroxide solution followed by incubation with PTEN antibody clone 138G8 (Cell Signaling, Beverly, MA) at a 1:800 dilution. Nuclear and cytoplasmic assays were performed. Percent positivity (0–100%) and level of intensity (0–3) were scored for each section. H-scores were then determined by multiplying the scores for intensity and percent positivity. PTEN high expressers were any non-zero H-score on nuclear or cytoplasmic assays. All other samples with an H-score of zero were classified as PTEN low expressers.

### Statistical analysis

The study employed stratified randomization to assign patients to two-parallel treatment arms. Patients were stratified by ER status. With 16 patients in each arm, the study achieves 77.7% power to detect a 45% improvement in the pCR + RCB I rate (0.40 vs. 0.85) at the 0.05 significance level using one-sided stratified Fisher’s exact test. To monitor against futility, Simon’s two-stage optimum design was used which required 3 responders among the first 5 experimental arm patients enrolled for accrual to continue. At an annual review, the DSMB noticed the stark contrast in efficacy between the two study arms and suggested closing the trial early for superiority. The trial closed with 14 patients on the experimental arm and 16 patients on the standard arm, achieving over 93% power to detect the observed 37.5% improvement (62.5% vs 100%) in the response rate (RCB-0 + RCB-1) using the stratified test at the 0.05 significance level. Baseline characteristics are reported as mean ± standard deviation for continuous variables and as counts and percentages for categorical factors. All patients who received at least 1 dose of treatment were included in the safety analysis. All analyses were conducted using SAS 9.4 (SAS Institute Inc., Cary, NC, USA) software with significance defined as *p* < 0.05.

## Results

### Patient demographics

A total of 30 patients were enrolled and evaluable. Fourteen patients were randomly assigned to the experimental arm (T-DM1 + lapatinib + nab-paclitaxel) and 16 to the standard arm (trastuzumab + pertuzumab + paclitaxel). The overall median patient age was 55 years (range 28–75). Sixty percent of patients were Caucasian and 30% were Hispanic. Ninety-three percent of cases were invasive ductal carcinoma. Tumor grade was almost equally divided between 2 and 3. Tumor stage was also almost equally divided between II and III in the experimental and standard arms (Table 1).

**Table 1:** Patient demographics and tumor characteristics by treatment arm

	Standard (*N* = 16)	Experimental (*N* = 14)	Total (*N* = 30)	*p* value
Median age, years (range)	57.2 (39.6–74.9)	53.1 (27.8–69.7)	54.7 (27.8–74.9)	0.42
Race/ethnicity				0.71
Caucasian	11 (68.8%)	7 (50.0%)	18 (60.0%)	
Hispanic	4 (25.0%)	5 (35.7%)	9 (30.0%)	
African American	0 (0.0%)	1 (7.1%)	1 (3.3%)	
Asian	1 (6.3%)	1 (7.1%)	2 (6.7%)	
Tumor type				0.21
Invasive ductal carcinoma	16 (100%)	12 (85.7%)	28 (93.3%)	
Invasive lobular carcinoma	0 (0.0%)	1 (7.1%)	1 (3.3%)	
Invasive mammary carcinoma	0 (0.0%)	1 (7.1%)	1 (3.3%)	
Stage				0.72
Stage II	7 (43.8%)	8 (57.1%)	15 (50.0%)	
Stage III	9 (56.3%)	6 (42.9%)	15 (50.0%)	
Hormone status				
ER negative	8 (50%)	6 (43%)	14 (46.6%)	
ER positive	8 (50%)	8 (57.1%)	16 (53.3%)	
Grade				0.27
Grade 2	5 (31.3%)	8 (57.1%)	13 (43.3%)	
Grade 3	11 (68.8%)	6 (42.9%)	17 (56.7%)	
Ki-67	50.5 (2--80)	55.9 (15–80)	53.2 (15–80)	0.54

### Efficacy

**Fig. 2:**
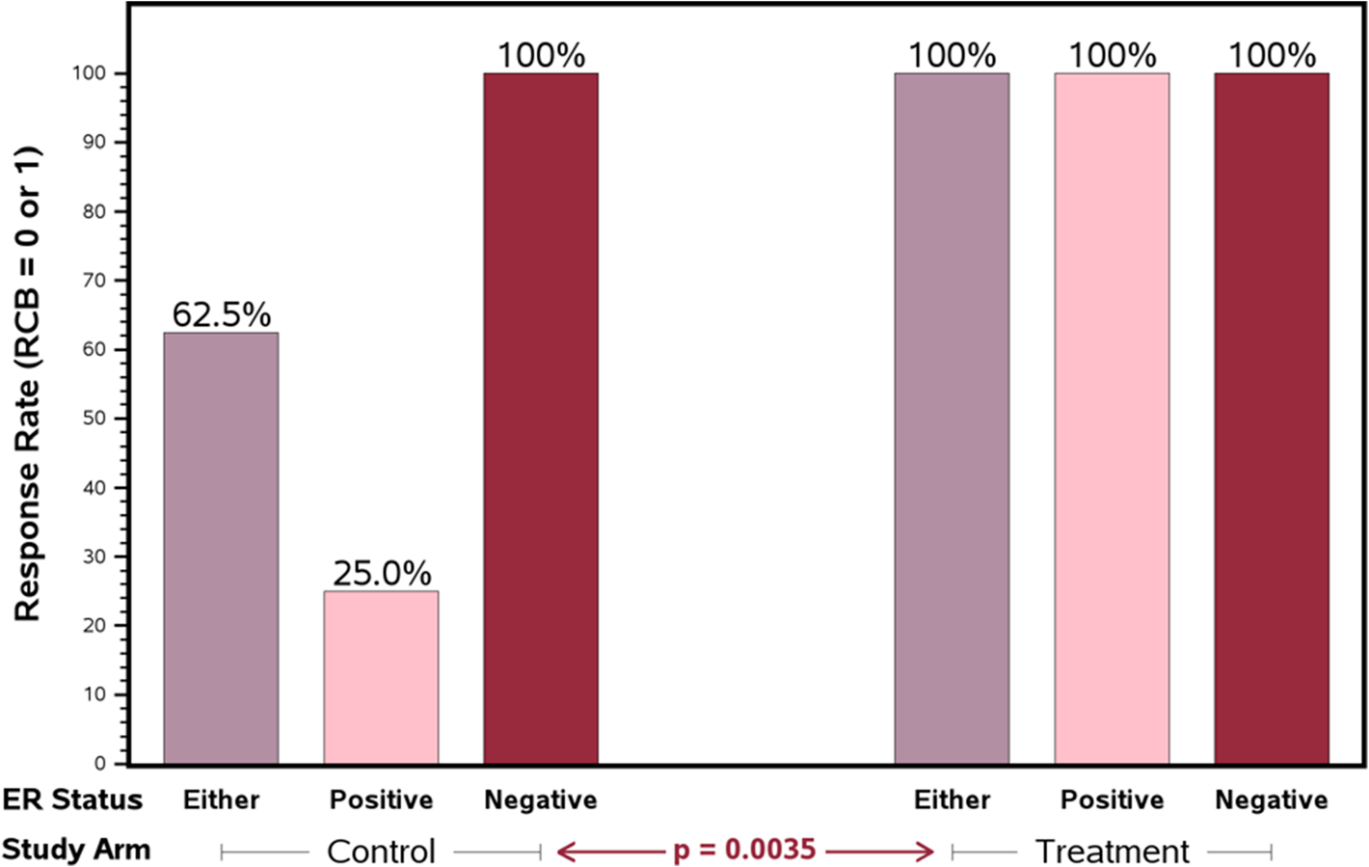
Pathologic response rate (RCB 0-I) variability by ER status

In the experimental arm, 100% of patients achieved RCB-0 or RCB-I at time of surgery (95% CI 78.4–100%). In the standard arm, 62.5% of patients achieved RCB-0 or RCB-I (95% CI 36.7–82.8%). The 37.5 percentage point improvement in response rate between the standard and experimental arm was statistically significant (*p* = 0.0035). In the ER-negative cohort, all patients achieved RCB-0 or RCB-I whether treated with the standard or experimental neoadjuvant protocol. Notably, in the ER-positive cohort, all patients in the experimental arm achieved RCB-0 or RCB-I compared with only 25% in the standard arm (*p* = 0.0035, Fig. 2). There was a trend towards an improved pCR (RCB = 0) rate between the experimental and standard arms (85.7% and 62.5%; *p* = 0.066; Table 2). On recommendation of the DSMB, this study was halted early because of the observed superior efficacy results on the experimental arm, particularly in the ER-positive subset.

**Table 2:** Pathologic findings at surgery

	Standard (*N* = 16)	Experimental (*N* = 14)	*p* value
RCB-0 and RCB-I	10 (62.5%)	14 (100.0%)	0.0035
ER negative	8	6	
ER positive	2	8	
RCB-II and RCB-III	6 (37.5%)	0 (0.0%)	
ER negative	0	0	
ER positive	6	0	
pCR	10 (62.5%)	12 (85.7%)	0.0660
ER negative	8	6	
ER positive	2	6	

RCB was pathologically determined from the (1) tumor bed dimensions, (2) cellularity of the invasive cancer, (3) size of largest nodal metastasis, and (4) number of positive lymph nodes. RCB was categorized as RCB-0 = no residual disease, RCB-I = minimal residual disease, RCB-II = moderate residual disease, and RCB-III = extensive residual disease [14]. RCB-0 was synonymous with pCR, indicating no residual disease present in the breast or axilla

As all patients in the experimental arm achieved RCB-0 or RCB-I, we evaluated changes in tumor size by MRI only in patients on the standard arm. The 6-week change in tumor size during targeted biologic window treatment was significantly different between eventual responders and non-responders based on the two-sided Wilcoxon rank-sum test (*p* = 0.0065, Table 3). Sixteen patients in total were enrolled in the standard arm, but 5 patients had incomplete imaging data so they were excluded. All patients in the experimental arm ultimately responded, so MRI data at 6 weeks was not relevant as a marker for response and thus was not reported.

**Table 3:** Change in tumor size on MRI after a 6-week biologic window for patients on standard treatment

Response	*N*	Mean shrinkage (cm)	Std. dev	Minimum (cm)	Maximum (cm)
No	6	− 0.13	0.45	− 1.0	0.3
Yes	5	2.58	1.88	0.2	4.9

### Safety

We categorized adverse events according to the NCI CTCAE version 4.03. Both treatment arms were similarly well tolerated. The overall incidence of all grade adverse events was similar between arms with no statistically observed difference. Common adverse events in both arms included elevated liver function tests, diarrhea, and fatigue. Two patients on the experimental arm experienced grade III/IV elevations in liver function tests versus none on the standard arm. This was reversible with treatment modification and ultimately not found to be a statistically significant difference between treatment arms. One patient on the experimental arm experienced a myocardial infarction, but this was not believed to be treatment-related (Table 4).

**Table 4:** Adverse events

	Grade	Standard (*N* = 16)	Experimental (*N* = 14)	*p* value
Liver function abnormalities	G I/II	9 (56.3%)	9 (64.3%)	0.72
	Grade III/IV	0 (0.0%)	2 (14.3%)	0.21
Fatigue	G I/II	8 (50.0%)	6 (42.9%)	0.73
	Grade III/IV	0 (0.0%)	1 (7.1%)	0.47
Diarrhea	G I/II	7 (43.8%)	7 (50.0%)	1.00
Neuropathy	G I/II	3 (18.8%)	3 (21.4%)	1.00
Rash	G I/II	3 (18.8%)	3 (21.4%)	1.00
Hypokalemia	G I/II	5 (31.3%)	2 (14.3%)	0.40
	Grade III/IV	1 (6.3%)	1 (7.1%)	1.00
Hypomagnesemia	G I/II	1 (6.3%)	0 (0.0%)	1.00
Mucositis	G I/II	1 (6.3%)	1 (7.1%)	1.00
Nail discoloration	G I/II	0 (0.0%)	1 (7.1%)	0.47
Skin discoloration	G I/II	0 (0.0%)	1 (7.1%)	0.47
Nausea	G I/II	3 (18.8%)	0 (0.0%)	0.23
Constipation	G I/II	0 (0.0%)	1 (7.1%)	0.47
Depression	G I/II	1 (6.3%)	1 (7.1%)	1.00
Anxiety	G I/II	1 (6.3%)	0 (0.0%)	1.00
Epistaxis	G I/II	0 (0.0%)	2 (14.3%)	0.21
Chest pain	G I/II	1 (6.3%)	0 (0.0%)	1.00
Myocardial infarction	G I/II	0 (0.0%	0 (0.0%)	1.00
	Grade III/IV/V	0 (0.0%)	1 (7.1%)	0.47
Musculoskeletal pain	G I/II	1 (6.3%)	0 (0.0%)	1.00
Breast pain	G I/II	1 (6.3%)	0 (0.0%)	1.00
Platelet count decreased	G I/II	0 (0.0%)	1 (7.1%)	0.47
Neutrophil count decreased	G I/II	0 (0.0%)	1 (7.1%)	0.47

### Biomarker exploratory analyses

Eleven patients had sufficient baseline tissue for HER2 subtype processing which was sent to Agendia for MammaPrint®, a 70-gene recurrence assay which predicts clinical outcome in women with early-stage breast cancer. Four of these samples resulted as non-HER2-type tumors: 3 luminal which were on the standard arm and 1 basal which was on the experimental arm. In this limited subset, there was no significant difference between the control and experimental arms whether analyzed by Satterthwaite’s *t* test (*p* = 0.1824) or by the Wilcoxon rank-sum test (*p* = 0.2474).

Twenty-five patient samples were evaluated for alterations in *PIK3CA* pathways. There was one *PIK3CA* H1047R mutation identified on Exon 20 in a patient on the experimental arm. Fifteen samples were sufficient for PTEN evaluation. Among ER-positive patients treated with standard treatment, PTEN low expressers were less likely to respond (0%, 0 of 2) when compared to PTEN high expressers (67%, 2 of 3).

## Discussion

Here, we report a multicenter randomized study where combination treatment with neoadjuvant T-DM1, lapatinib, and nab-paclitaxel was highly effective with adequate tolerability and similar adverse events when compared to neoadjuvant trastuzumab, pertuzumab, and paclitaxel. Though historically pCR in ER-positive patients has been more difficult to obtain [4, 6, 15], responses in the experimental arm were observed in both ER-negative and ER-positive patients. A phase Ib/IIa study of neoadjuvant T-DM1, pertuzumab, and docetaxel reported a pCR rate of 60.6% overall. The pCR rate in the ER-positive, HER2-positive cohort was 54.2% [16]. The recent prospective, neoadjuvant phase II ADAPT study noted that ER-positive patients achieved a higher pCR when T-DM1 was utilized ± endocrine therapy when compared to trastuzumab and endocrine therapy (41% vs 6.7%; *p* < 0.001) [17]. Yet, in the randomized phase 3 Kristine study, dual blockade with T-DM1 and pertuzumab led to pCR in 44.4% of women while standard of care trastuzumab, pertuzumab, and chemotherapy yielded a significantly higher pCR rate. Specifically, in the ER-positive cohort, the pCR rate was 37.9% with T-DM1 and pertuzumab vs. 44.8% with chemotherapy [18]. We report high pathologic responses with T-DM1 and lapatinib dual blockade along with chemotherapy, especially in the ER-positive HER2-positive cohort. The molecular mechanism for this observation is unclear but could be related to the dual mechanism of T-DM1 as a chemotherapy agent as well as HER2-targeting drug. Future studies are merited to better elucidate the HER2 synergistic mechanism of this regimen as well as apply this regimen to a larger patient population. With additional study, this protocol may provide a valuable, more efficacious option for early-stage ER-positive HER2-positive patients who are typically more refractory to treatment.

One of the exploratory objectives of this study was to determine the molecular-genetic determinants for T-DM1 and lapatinib combined dual blockade. Of the available samples, Mammaprint® HER2 heterogeneity was not significantly different in both treatment arms. ER status was well-matched as were other potential confounding variables. All patients on the experimental arm responded, even the HER2/luminal subtype. Previously, we demonstrated through neoadjuvant clinical trials that activated PI3K pathway (somatic *PIK3CA* mutations and loss of PTEN) was associated with resistance to trastuzumab as well as trastuzumab combined with lapatinib [7]. However, other recent studies have shown that these patients with activated PI3K pathway may benefit from T-DM1 [19]. In our study, response in the experimental arm also occurred regardless of PTEN status. Among ER-positive patients treated with standard treatment, PTEN low expressers were less likely to respond than PTEN high expressers. Though conclusions in this area are limited by small patient numbers, baseline PTEN low expression appeared to select patients who do not respond to treatment. This is consistent with the published literature [7].

## Conclusion

We report a highly effective neoadjuvant regimen of T-DM1, lapatinib, and nab-paclitaxel where ER status and genetic molecular subtypes do not appear to predict for resistance. Safety is preserved on this regimen with similar frequency of adverse events noted when compared to standard of care. The observed efficacy of T-DM1 in this setting, especially in ER-positive patients, in combination with other targeted agents or anti-estrogens remains to be explored and confirmed in further clinical studies.

## Data Availability

The datasets used during the current study are available from the corresponding author on reasonable request.

## Abbreviations

CARE: Consortium for the Advancement of Research Excellence
DSMB: Data Safety and Monitoring Board
ER: Estrogen receptor
FFPE: Formalin-fixed, paraffin-embedded
HER2: Human epidermal growth factor receptor
MRI: Magnetic resonance imaging
NCI CTCAE: National Cancer Institute Common Terminology Criteria for Adverse Events
pCR: Pathologic complete response
PI3K: Phosphoinositide 3-kinase
PTEN: Phosphatase and tensin homolog
RCB: Residual cancer burden
T-DM1: Ado-trastuzumab emtansine

## References

[1] Hurvitz L, Dirix L, Kocsis J, Bianchi G, Lu J, Vinholes J, Guardino E, Song C, Tong B, Ng V, Chu Y, Perez E (2013). Phase II randomized study of trastuzumab emtansine versus trastuzumab plus docetaxel in patients with human epidermal growth factor receptor-2 positive metastatic breast cancer. J Clin Oncol.

[2] Barok M, Joensuu H, Isola J (2014). Trastuzumab emtansine: mechanisms of action and drug resistance. Breast Cancer Res.

[3] Baselga J, Bradbury I, Eidtmann H, Di Cosimo S, de Azambuja E, Aura C, Gómez H, Dinh P, Fauria K, Van Dooren V, Aktan G, Goldhirsch A, Chang T, Horváth Z, Coccia-Portugal M, Domont J, Tseng L, Kunz G, Sohn J, Semiglazov V, Lerzo G, Palacova M, Probachai V, Pusztai L, Pusztai L, Untch M, Gelber R, Piccart-Gebhart M (2012). Lapatinib with trastuzumab for HER2-positive early breast cancer (NeoALTTO): a randomised, open-label, multicentre, phase 3 trial. Lancet..

[4] Gianni L, Pienkowski T, Im Y, Roman L, Tseng L, Liu M, Lluch A, Staroslawska E, Haba-Rodriguez J, Im S, Pedrini J, Poirier B, Morandi P, Semiglazov V, Srimuninnimit V, Bianchi G, Szado T, Ratnayake J, Ross G, Valagussa P (2012). Efficacy and safety of neoadjuvant pertuzumab and trastuzumab in women with locally-advanced, inflammatory or early HER2-positive breast cancer (NeoSphere): a randomized, open-label, phase 2 trial. Lancet Oncol.

[5] Dang C, Iyengar N, Datko F, D'Andrea G, Theodoulou M, Dickler M, Goldfarb S, Lake D, Fasano J, Fornier M, Gilewski T, Modi S, Gajria D, Moynahan M, Hamilton N, Patil S, Jochelson M, Norton L, Baselga J, Hudis C (2015). Phase II study of paclitaxel given once per week along with trastuzumab and pertuzumab in patients with human epidermal growth factor receptor 2-positive metastatic breast cancer. J Clin Oncol.

[6] Carey L, Berry D, Cirrincione C, Barry W, Pitcher B, Harris L, Ollila D, Krop I, Henry N, Weckstein D, Anders C, Singh B, Hoadley K, Iglesia M, Cheang M, Perou C, Winer E, Hudis C (2016). Molecular heterogeneity and response to neoadjuvant human epidermal growth factor receptor 2 targeting in CALGB 40601, a randomized phase III trial of paclitaxel plus trastuzumab with or without lapatinib. J Clin Oncol.

[7] Rimawi M, De Angelis C, Contreras A, Pareja F, Geyer F, Burke K, Herrera S, Wang T, Mayer I, Forero A, Nanda R, Goetz M, Chang J, Krop I, Wolff A (2018). Low PTEN levels and PIK3CA mutation predict resistance to neoadjuvant lapatinib and trastuzumab without chemotherapy in patients with HER2 over-expressing breast cancer. Breast Cancer Res Treat.

[8] Dave B, Migliaccio I, Gutierrez M, Wu M, Chamness G, Wong H, Narasanna A, Chakrabarty A, Hilsenbeck S, Huang J, Rimani M, Schiff R, Arteaga C, Osborne C, Chang J (2011). Loss of phosphatase and tensin homolog or phosphoinositol-3 kinase activation and response to trastuzumab or lapatinib in human epidermal growth factor receptor 2-overexpressing locally advanced breast cancers. J Clin Oncol.

[9] Davis N, Sokolosky M, Stadelman K, Abrams S, Libra M, Candido S, Nicoletti F, Polesel J, Maestro R, D’Assoro A, Drobot L, Rakus D, Gizak A, Laidler P, Dulinska-Litewka J, Basecke J, Mijatovic S, Maksimovic-Ivanic D, Montalto G, Cervello M, Fitzgerald T, Demidenko Z, Martelli A, Cocco L, Steelman L, McCubrey J (2014). Deregulation of the EGFR/PI3K/PTEN/Akt/mTORC1 pathway in breast cancer: possibilities for therapeutic intervention. Oncotarget..

[10] Verma S, Miles D, Gianni L, Krop I, Welslau M, Baselga J, Pegram M, Oh D, Diéras V, Guardino E, Fang L, Lu M, Olsen S, Blackwell K (2012). Trastuzumab emtansine for Her2-positive advanced breast cancer. N Engl J Med.

[11] Patel T, Ensor J, Rodriguez A, Belcheva A, Darcourt J, Niravath P, Kuhn J, Kaklamani V, Li X, Boone T, Chang J (2018). Phase Ib study of trastuzumab emtansine (TDM1) in combination with lapatinib and nab-paclitaxel in metastatic HER2-neu overexpressed breast cancer patients: STELA results.

[12] Furlanetto J, Jackisch C, Untch M, Schneeweiss A, Schmatloch S, Aktas B, Denkert C, Wiebringhaus H, Kummel S, Warm M, Paepke S, Just M, Hanusch C, Hackmann J, Blohmer J, Clemens M, Costa S, Gerber B, Nekljudova V, Loibl S, Minckwitz G (2017). Efficacy and safety of nab-paclitaxel 125mg/m2 and nab-paclitaxel 150mg/m2 compared to paclitaxel in early high-risk breast cancer. Results from the neoadjuvant randomized GeparSepto study (GBG 69). Breast Cancer Res Treat.

[13] Wolff A, Hammond M, Hicks D, Dowsett M, McShane L, Allison K, Allred D, Bartlett J, Bilous M, Fitzgibbons P, Hanna W, Jenkins R, Mangu P, Paik S, Perez E, Press M, Spears P, Vance G, Viale G, Hayes D (2013). Recommendations for human epidermal growth factor receptor 2 testing in breast cancer: American Society of Clinical Oncology/College of American Pathologists Clinical Practice Guideline Update. J Clin Oncol.

[14] Symmans W, Peintinger F, Hatzis C, Rajan R, Kuerer H, Valero V, Assad L, Poniecka A, Hennessy B, Green M, Buzdar A, Singletary S, Hortobagyi G, Pusztai L (2007). Measurement of residual cancer burden to predict survival after neoadjuvant chemotherapy. J Clin Oncol.

[15] Rimawi M, Mayer I, Forero A, Nanda R, Goetz M, Rodriguez A, Pavlik A, Wang T, Hilsenbeck S, Gutierrez C, Schiff R, Osborne C, Chang J (2013). Multicenter phase II study of neoadjuvant lapatinib and trastuzumab with hormonal therapy and without chemotherapy in patients with human epidermal growth factor receptor 2-overexpressing breast cancer: TBCRC 006. J Clin Oncol.

[16] Martin M, Fumoleau P, Dewar J, Albanell J, Limentani S, Campone M, Chang J, Patre M, de Haas ASS, Xu J, Garcia-Saenz J (2016). Trastuzumab emtansine (T-DM1) plus docetaxel with or without pertuzumab in patients with HER2-positive locally advanced or metastatic breast cancer: results from a phase Ib/IIa study. Ann Oncol.

[17] Harbeck N, O. Gluz, M. Christgen, M. Braun, S. Kuemmel, C. Schumacher, J. Potenberg, S. Kraemer, A. Kleine-Tebbe, D. Augustin, B. Aktas, H. Forstbauer, J. Tio, C. Liedtke, R. Kates, R. Wuerstlein, S. de Haas, A. Kiermaier, H. Kreipe, U. Nitz, editor Final analysis of WSG-ADAPT HER2+/HR+ phase II trial: Efficacy, safety, and predictive markers for 12-weeks of neoadjuvant TDM1 with or without endocrine therapy versus trastuzumab+endocrine therapy in HER2-positive hormone-receptor-positive early breast cancer. CTRC-AACR San Antonio Breast Cancer Symposium; 2015; San Antonio.

[18] Hurvitz S, Martin M, Symmans W, Jung K, Huang C, Thompson A, Harbeck N, Valero V, Stroyakovskiy D, Wildiers H, Campone M, Boileau J, Beckmann M, Afenjar K, Fresco R, Helms H, Xu J, Lin Y, Sparano J, Slamon D (2018). Neoadjuvant trastuzumab, pertuzumab, and chemotherapy versus trastuzumab emtansine plus pertuzumab in patients with HER2-positive breast cancer (KRISTINE): a randomized, open-label, multicentre, phase 3 trial. Lancet Oncol..

[19] Baselga J, Phillips G, Verma S, Ro J, Huober J, Guardino A, Samant M, Olsen S, de Haas S, Pegram M (2016). Relationship between tumor biomarkers and efficacy in EMILIA, a phase III study of trastuzumab emtansine in Her2-positive metastatic breast cancer. Clin Cancer Res.

